# Production of the 2,5-Furandicarboxylic Acid Bio-Monomer From 5-Hydroxymethylfurfural Over a Molybdenum-Vanadium Oxide Catalyst

**DOI:** 10.3389/fchem.2022.853112

**Published:** 2022-03-14

**Authors:** Jian Liu, Sha Wen, Fei Wang, Xiaoting Zhu, Zhijuan Zeng, Dulin Yin

**Affiliations:** National and Local Joint Engineering Laboratory for New Petro-chemical Materials and Fine Utilization of Resources, Key Laboratory of the Assembly and Application of Organic Functional Molecules of Hunan Province, Hunan Normal University, Changsha, China

**Keywords:** biomass, 5-hydroxymethylfurfural, 2, 5-furandicarboxylic acid, selective oxidation, no-noble metal catalyst

## Abstract

2, 5-Furandicarboxylic acid (FDCA) is an important bio-monomer that can potentially replace terephthalic acid to synthesize degradable polyesters. Efficient selective oxidation of biomass-based 5-hydroxymethylfurfural (HMF) to FDCA has been a significant but challenging work in the past decades. In this study, a novel molybdenum-vanadium oxide (Mo-V-O) catalyst was prepared by a simple method and showed excellent catalytic activity for converting HMF to FDCA. A high FDCA selectivity of 94.5 and 98.2% conversion of HMF were achieved under the optimal conditions with tert-butyl hydroperoxide as the oxidant. FT-IR, SEM, XRD and TG were applied to investigate the properties of Mo-V-O catalyst. After fitting experimental data with the first-order kinetics equation, the evaluated apparent activation energies of HMF oxidation were obtained. The experimental design and study were carried out by response surface methodology (RSM) to test the effects of reaction conditions on the catalytic process.

## 1 Introduction

Owing to the exhaustion of fossil resources and the aggravation of environmental problems, many efforts have been made to exploit sustainable and environmentally friendly alternatives (Gao et al., 2018; Pal and Saravanamurugan, 2020). As such, the excavation of renewable resources that can be used to produce extensive commodity chemicals is of great significance (Zhao et al., 2020). In this context, biomass is the most abundant renewable resource, and is considered a potential material for industrial production (Guo et al., 2020). Hence, the exploitation of biomass represents a significant step towards the goal of sustainable development of natural resources (Lin et al., 2021). In addition, through their depolymerization, biopolymers derived from biomass can be further converted into high-value-added platform chemical compounds for use in subsequent applications.

5-Hydroxymethylfurfural (HMF) which originates from cellulose or cellulose-derived carbohydrates, has been identified as a platform compound that can be intensively processed into 2,5-diformylfuran (DFF), 5-hydroxymethyl-2-furancarboxylic acid (HFCA), 5-formyl-2-furancarboxylic acid (FFCA), and 2,5-furandicarboxylic acid (FDCA) (Pal and Saravanamurugan, 2020; Hong et al., 2019a; Hong et al., 2019b). More specifically, the oxidative transformations of HMF are key routes toward a wide variety of chemicals and biofuels (Gao et al., 2020; Hong et al., 2021). In particular, FDCA, which is derived from the successive oxidation of HMF, is a substitute for petroleum-derived terephthalic acid, which can be employed in the preparation of polyethylene furanoate (PEF) to ultimately alleviate the shortage of resources and relieve environmental stress (Tirsoaga et al., 2020).

The most common preparation of FDCA is carried out under basic conditions, which complicates downstream separation, increases the number of added reagents, and generates inorganic salts (Hayashi et al., 2019). Therefore, the development of a basic-free system is considered of particular importance. Furthermore, the high costs of noble metals renders these catalysts less attractive, and hinders scale-up industrialization (Zhou et al., 2018). Alternatively, the advantages of transition metals are well known; however, vanadium, which is commonly used in the oxidation of HMF, undergoes a loss of its active components during the reaction, and so tends to be employed in combination with other elements. (Navarro et al., 2009; Rodikova and Zhizhina, 2020). In this context, Zhao *et al.* synthesized a catalyst based on vanadium dioxide (VO_2_)-embedded mesoporous carbon spheres (V-CS) using a facile hydrothermal method, which produced DFF in a yield of 99.0% (Zhao et al., 2018). In addition, Lai *et al.* prepared a new type of magnetic vanadium-based catalyst, namely NH_4_·V_3_O_8_/Fe_3_O_4_ (M-V-O), which gave a 95.5% conversion of HMF and a 82.9% selectivity toward DFF (Lai et al., 2020). More recently, Wang *et al.* used a novel crystalline Mo-V-O oxide as a heterogeneous catalyst in the liquid phase with molecular oxygen for the selective oxidation of alcohols (Wang and Ueda, 2009), while Rasteiro *et al.* investigated the one-step oxydehydration of glycerol to acrylic acid over molybdenum and vanadium mixed oxides, achieving a selectivity of 33.5% towards acrylic acid and a 100% conversion of glycerol (Rasteiro et al., 2017). Therefore, Mo-V-O, which contains non-noble metals, appears to be suitable for use as a catalyst for the oxidation of HMF to FDCA without the requirement for a base.

Thus, we herein report the development of a simple and feasible method for preparing a catalyst for the oxidation of HMF to FDCA in the absence of base. During this study, a range of reaction parameters, such as the temperature, reaction time, HMF/TBHP molar ratio, and catalyst amount, are examined and optimized. A series of characterization methods are then applied to investigate the catalyst, and a kinetic study and response surface methodology (RSM) are also carried out.

## 2 Materials and Methods

### 2.1 Materials

5-Hydroxymethylfurfural (97.0%) and *tert*-butyl hydroperoxide (TBHP) (70.0%) were purchased from Shanghai Macklin Biochemical Co., Ltd (Shanghai, China). 2,5-Dimethylfuran (≥98.0%) was purchased from Shanghai D&B Biological Science and Technology Co., Ltd (Shanghai, China) (NH_4_)_6_Mo_7_O_24_·4H_2_O, C_6_H_8_O_7_·H_2_O, and *tert*-butanol (TBA) (≥99.0%) were purchased from Sinopharm Chemical Reagent Co., Ltd (Shanghai, China). NH_4_VO_3_ (≥99.0%) was purchased from Tianjin Guangfu Technology Development Co., Ltd (Tianjin, China). V_2_O_5_ was purchased from Tianjin Guangfu Technology Development Co., Ltd. MoO_3_ was purchased from Tianjin Guangfu Technology Development Co., Ltd.

### 2.2 Catalyst Preparation

Mo-V-O was synthesized according to the following procedure (NH_4_)_6_Mo_7_O_24_·4H_2_O (0.003 mol) and NH_4_VO_3_ (0.018 mol) were mixed in deionized water (50 ml) and heated for 30 min at 80°C. Subsequently, C_6_H_8_O_7_·H_2_O was slowly added and after stirring for 12 h, the reaction mixture was dried for 12 h in an oven. The mixture was then ground and calcined at 500°C in a muffle furnace for 5 h. The prepared samples are denoted as Mo-V-O.

### 2.3 Characterization

The synthesized samples were characterized using Fourier transform infrared spectroscopy (FT-IR). The spectra of the catalysts were collected using the KBr pellet technique on a Nicolet 370 (Thermo Nicolet, American) infrared spectrophotometer in the wavenumber range of 400–4,000 cm^−1^. Thermogravimetry-differential thermal analysis (TG-DTG, Germany) curves were recorded under a N_2_ flow on a Netzsch Model STA 409 PC instrument (Mark, American) using *α*-Al_2_O_3_ as the standard material. Data were recorded from room temperature to 800°C at a heating rate of 10°C/min. X-ray diffraction (XRD) studies were performed using a Bruker diffractometer with Cu Kα radiation to survey the crystal structure over a 2θ range of 10–80°. Scanning electron microscopy (SEM) images were collected using a Zeiss Sigma 300 microscope (Carl Zeiss, Germany).

### 2.4 The Oxidation of HMF

HMF (0.5 mmol) was dissolved in 5 ml TBA while Mo-V-O (30 mg) was added. In the case of adding oxidant TBHP (5 mmol), the reaction carried out at 80 °C in round-bottom flask. The reactor was cooled to room temperature. The reaction mixture was analyzed by Agilent 1260 HPLC system, equipped with Venusil XBP C18 chromatographic column (4.6 × 250 mm, 5 μm, Phenomenex, United States) and a UV–Vis (280 nm) detector was used to analysis product. The quantification was carried out using the external standard method. The mobile phase was constituted of acetic acid solution (0.1 wt%) and acetonitrile in a volume ratio of 95: 5, and the samples were identified at a rate of 0.7 ml/min at 30°C. The equations were as follows:

HMF conversion (%)=(1- moles of HMF/moles of HMF added) × 100
FDCA selectivity (%) = moles of FDCA/(moles of HMF added - moles of HMF) ×100

## 3 Results and Discussion

### 3.1 Catalyst Characterization

#### 3.1.1 Infrared Spectra of Mo-V-O

**FIGURE 1:**
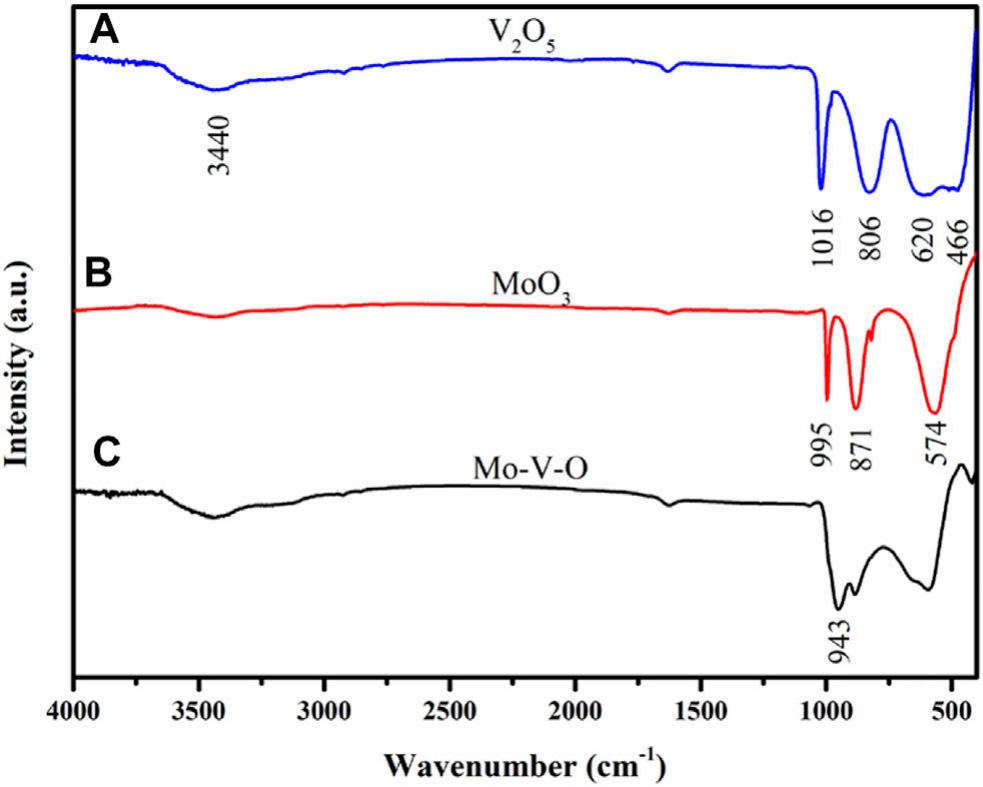
FT-IR spectra of the samples: **(A)** V_2_O_5_, **(B)** MoO_3_, and **(C)** Mo-V-O.

The FTIR spectra of the samples are shown in Figure 1, where the broad peak at 3,440 cm^−1^ corresponds to the H-OH bending vibration of physically adsorbed water. In addition, the characteristic peaks of V_2_O_5_ at 1,016 and 806 cm^−1^ originate from the symmetric stretching vibrations of V-O and V=O, while the peaks at 620 and 466 cm^−1^ could be attributed to the symmetric stretching vibration and asymmetric stretching vibration of V-O-V, respectively (Zhang et al., 2016). Furthermore, strong peaks at 995, 871, and 574 cm^−1^ can also be seen in the FT-IR spectra, which could be associated with MoO_3_, wherein the peak at 943 cm^−1^ arises from Mo-O-V, thereby confirming the formation of Mo-V-O (Guan et al., 2008).

#### 3.1.2 XRD Patterns of Mo-V-O Containing Various Mo/V Molar Ratios

**FIGURE 2:**
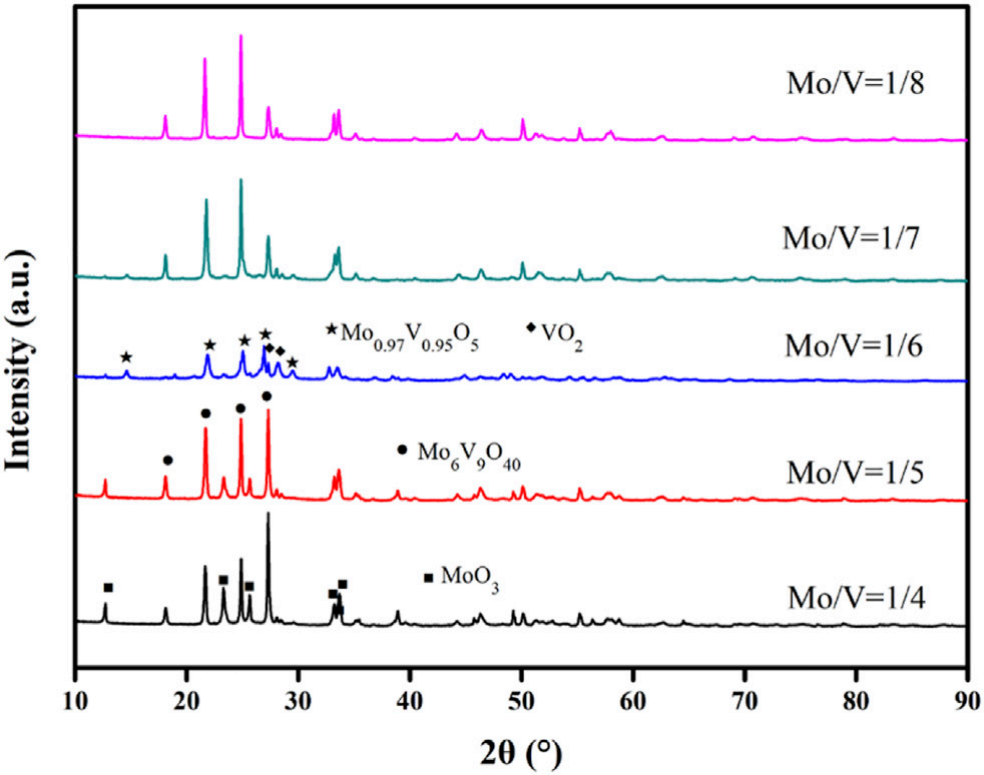
XRD patterns of the samples.

XRD analysis was performed to determine the crystal structure of the catalyst. Thus, the XRD spectra of the samples are presented in Figure 2, and show the characteristic reflections of MoO_3_ (JCPDF 05-0508), Mo_6_V_9_O_40_ (JCPDF 34-0527), VO_2_ (JCPDF 33-1,440), and Mo_0.97_V_0.95_O_5_ (JCPDF 50-0535), as indicated in the figure. It can be seen that upon increasing the Mo/V molar ratio, the characteristic peak intensities for MoO_3_ at 2*θ* = 12.7, 23.3, and 25.5° decreased. The molar ratio that gave the optimal catalytic effect was determined to be 1/6, in which the characteristic peaks at 2*θ* = 14.6, 21.8, 25.0, and 26.9° were assigned to Mo_0.97_V_0.95_O_5_. Moreover, the characteristic peaks at 2*θ* = 27.3 and 28.2° were attributed to VO_2_, which were also visible at a Mo/V molar ratio of 1/6.

#### 3.1.3 TG Curve of Mo-V-O

**FIGURE 3:**
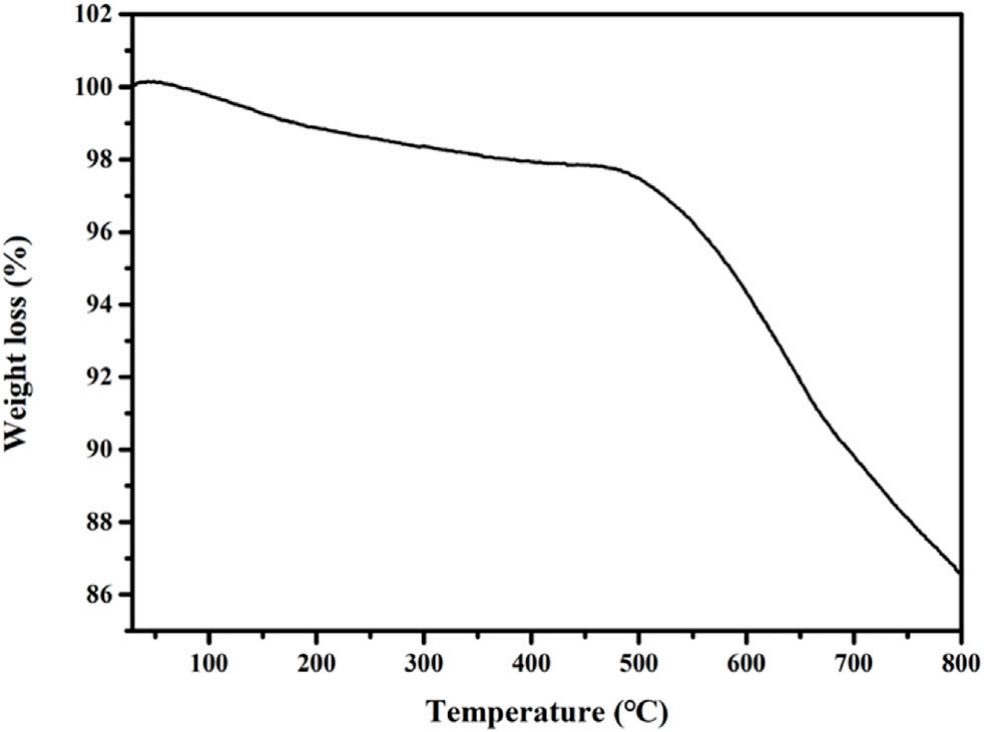
TG pattern of Mo-V-O.

The stability of a catalyst is a key performance parameter, and so we investigated the thermal stability, which is an indicator of the catalyst stability, using the TG approach. As shown in Figure 3, the TG thermogram shows that the catalyst underwent two major mass loss stages. The first mass loss stage at ∼190°C was attributed to the thermal dehydration of the catalyst, while the second and main mass-loss stage was ascribed to the decomposition of metal oxides at ∼660°C.

#### 3.1.4 SEM Images of Mo-V-O

**FIGURE 4:**
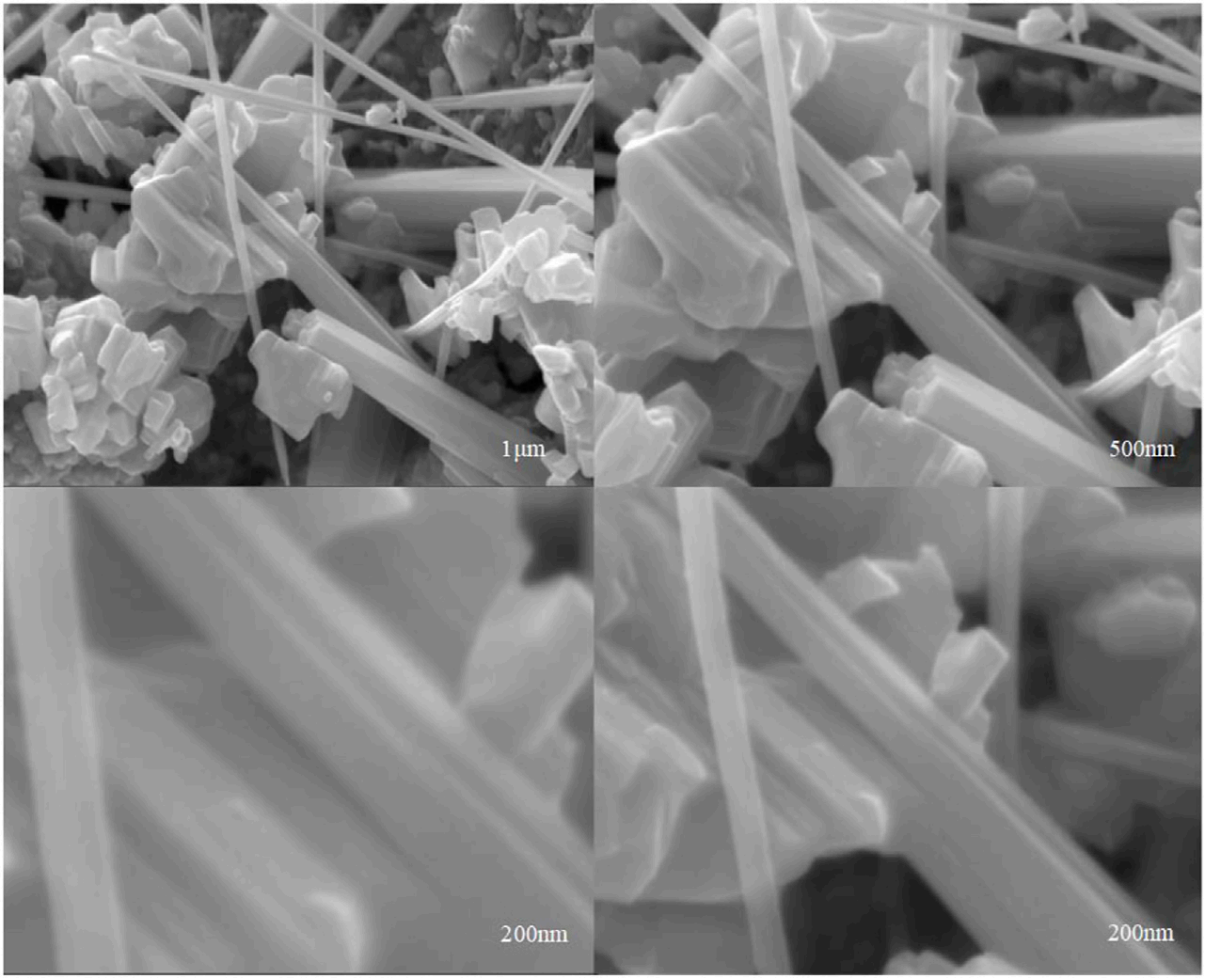
SEM images of Mo-V-O.

In order to obtain morphology information of the catalyst, the SEM was applied and shown in Figure 4. It can be seen that the catalyst appear nanorods morphology, and bulk-like morphology which formed by stacking of flake crystals. Most of Mo-V-O showed bulk-like structure which exhibited an irregular size, and the flake-like structure is observed clearly. Furthermore, the dimensions of nanorods were less consistent and comprised of different sizes showing that Mo-V-O material possessed different compositions, which is consistent with XRD.

### 3.2 Effects of Different Mo/V Molar Ratio on the Oxidation of HMF by Mo-V-O

**FIGURE 5:**
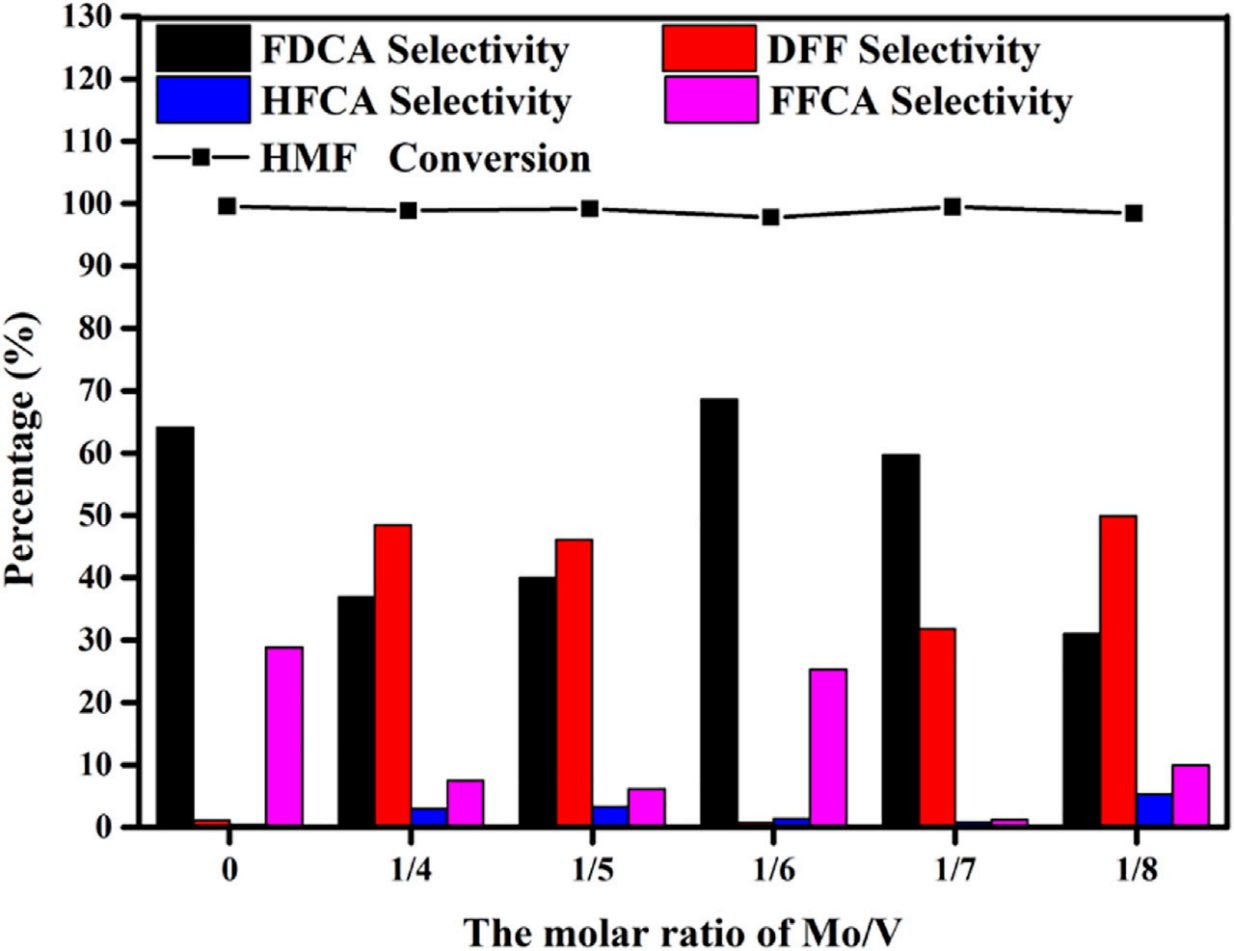
Effects of the Mo/V molar ratios on the catalytic oxidation of HMF. Reaction conditions:0.5 mmol HMF, 5 mmol TBHP, 30 mg Mo-V-O catalyst, 5 ml TBA, 10 h, 80°C.

Figure 5 displayed the products distribution of HMF converting to FDCA by pure V_2_O_5_ and the different molar ratio of Mo/V. In the presence of TBHP, V_2_O_5_ exhibits the ability of HMF oxidation to FDCA. However, there was only liquid without any catalyst after reaction indicating the instability of vanadium. Moreover, the composition of catalyst is an important factor affecting the performance of catalyst. As the changed Mo/V molar ratio from 1/4 to 1/6, FDCA selectivity varied from 36.9 to 68.6% at the same condition. Further adjusted the Mo/V molar ratio, a selective decline appeared. The above different catalytic effects indicate that the composition of different catalysts may be different, which is consistent with the XRD results.

### 3.3 Influence of the Mo-V-O Amount on the Oxidation of HMF

**FIGURE 6:**
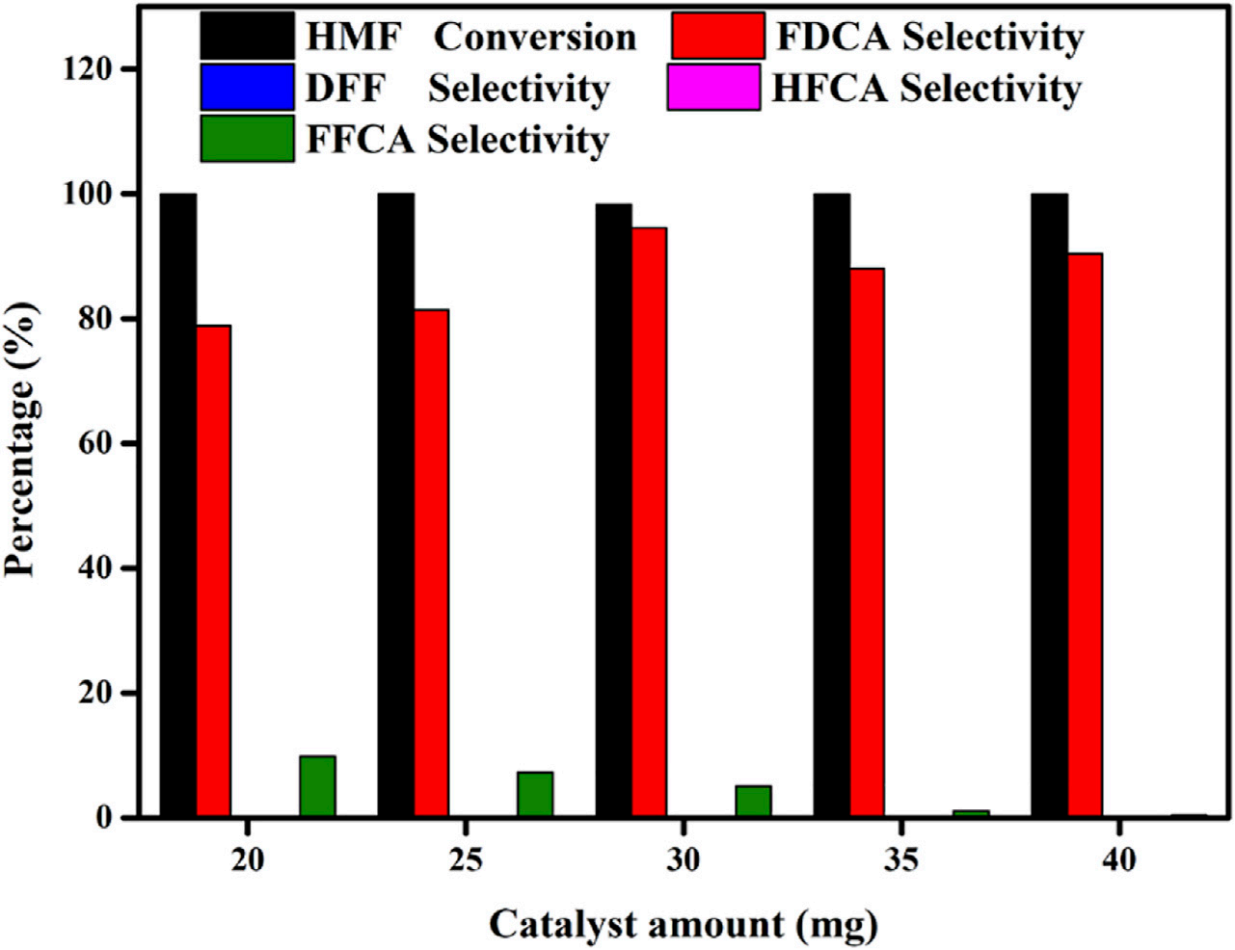
Effects of the amount of Mo-V-O catalyst on the oxidation of HMF. Reaction conditions:0.5 mmol HMF, 10 mmol TBHP, 5 ml TBA, 80°C, 10 h.

Generally, the number of active sites directly affects the catalytic performance. Thus, to optimize the catalytic performance, we examined the effect of the catalyst dosage at values ranging from 20 to 40 mg, and the results are presented in Figure 6. Upon increasing the catalyst loading, the number of active sites and the contact area between the catalyst and the substrate also increased, thereby influencing the reaction outcome. Interestingly, the HMF conversion did not change significantly with an increase in the catalyst dosage, indicating that even at a low catalyst dosage, the HMF conversion was adequate. However, the relationship between the catalyst dosage and the number of active sites was found to affect the selectivity toward FDCA. More specifically, as the catalyst dosage was initially increased from 20 to 30 mg, the selectivity toward FDCA increased from 87.6 to 94.5%. However, upon further increasing the amount of catalyst to 40 mg, the selectivity toward FDCA decreased, likely due to the fact than an excess of catalyst decreased the probability of effective contact between the catalyst and the substrate. This suggests that a 30 mg catalyst loading was sufficient for the conversion of HMF to FDCA.

### 3.4 Influence of the HMF/TBHP Molar Ratio on the Oxidation of HMF

**FIGURE 7:**
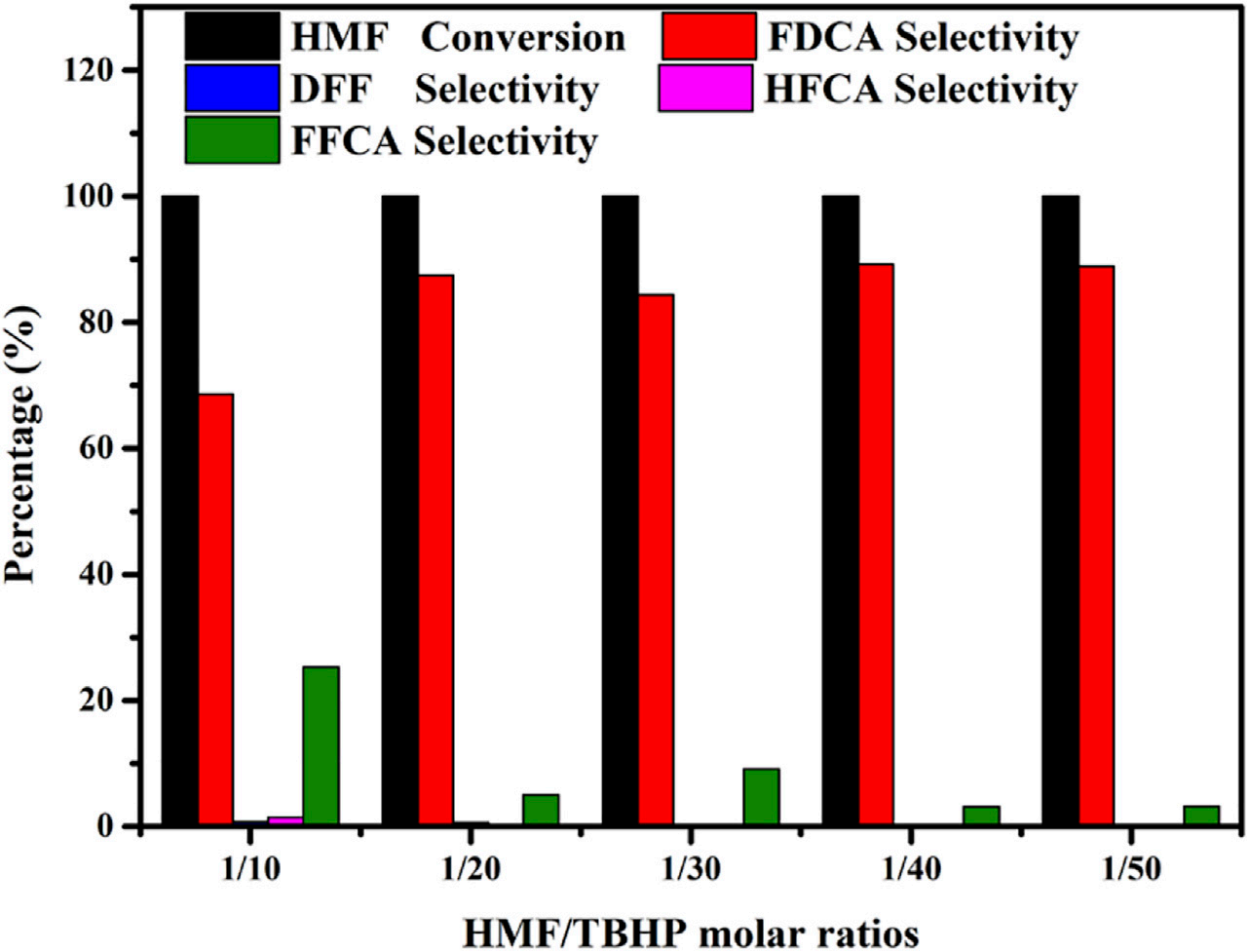
Effects of the HMF/TBHP molar ratio on the reaction selectivity. Reaction conditions:0.5 mmol HMF, 30 mg catalyst, 5 ml TBA, 4 h, 80°C.

**SCHEME 1:**
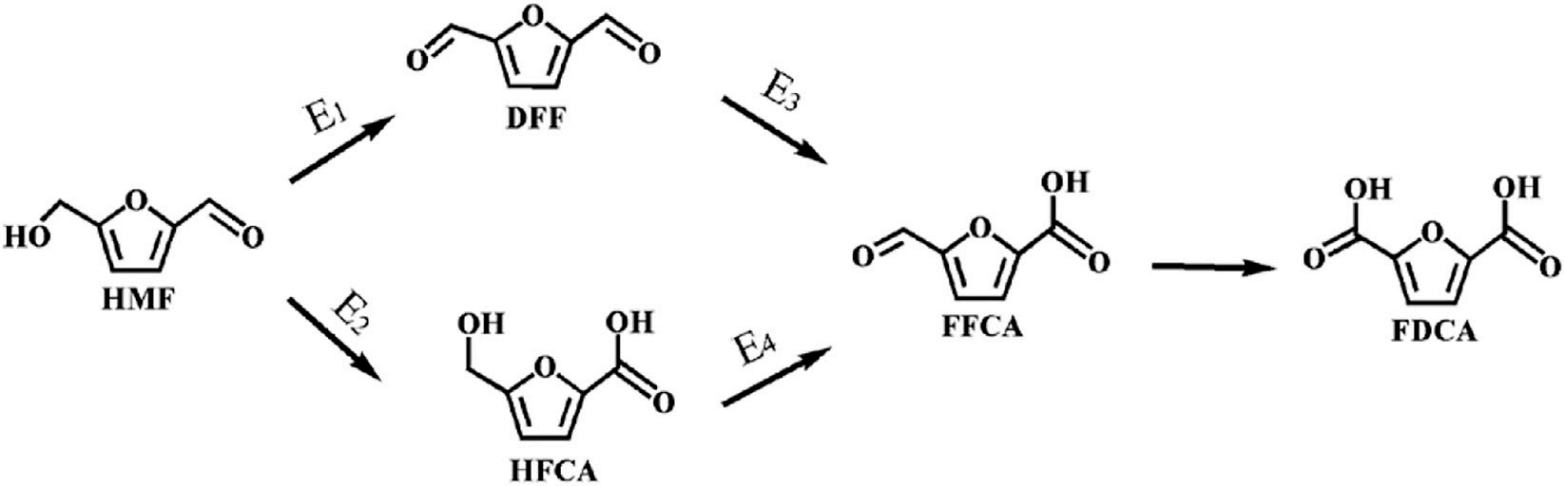
Reaction pathway of the aerobic oxidation of HMF to FDCA over Mo-V-O.

Since TBHP is employed as an oxidant in the HMF-to-FDCA pathway, different amounts of TBHP can also produce diverse catalytic performances, and thus the amount of TBHP is a crucial parameter. Hence, to gain further insight into the effect of TBHP on the oxidation of HMF into FDCA over Mo-V-O, different HMF/TBHP molar ratios were investigated. As shown in Figure 7, the conversion of HMF and the corresponding selectivity towards different oxidation products were found to vary under different HMF: TBHP ratios. More specifically, the selectivity toward FDCA increased substantially from 5.9 to 62.7% as the HMF/TBHP molar ratio was increased from 1/10 to 1/50, thereby indicating that TBHP was required for the selective oxidation of HMF. However, upon increasing this ratio to 1/20, the conversion of HMF to FDCA reached 97.4%, beyond which point, only minor increases were observed. Based on the products detected following this reaction, it was apparent that the aldehyde and hydroxyl groups present on the furan ring of HMF were oxidized to their corresponding intermediates, which further afforded FDCA from FFCA, as outlined in Scheme 1. Thus, from both economic and environmental perspectives, an HMF/TBHP molar ratio of 1/20 was considered optimal for the conversion of HMF, and so was selected for further experiments.

### 3.5 Influence of the Reaction Time on Oxidation of HMF

**FIGURE 8:**
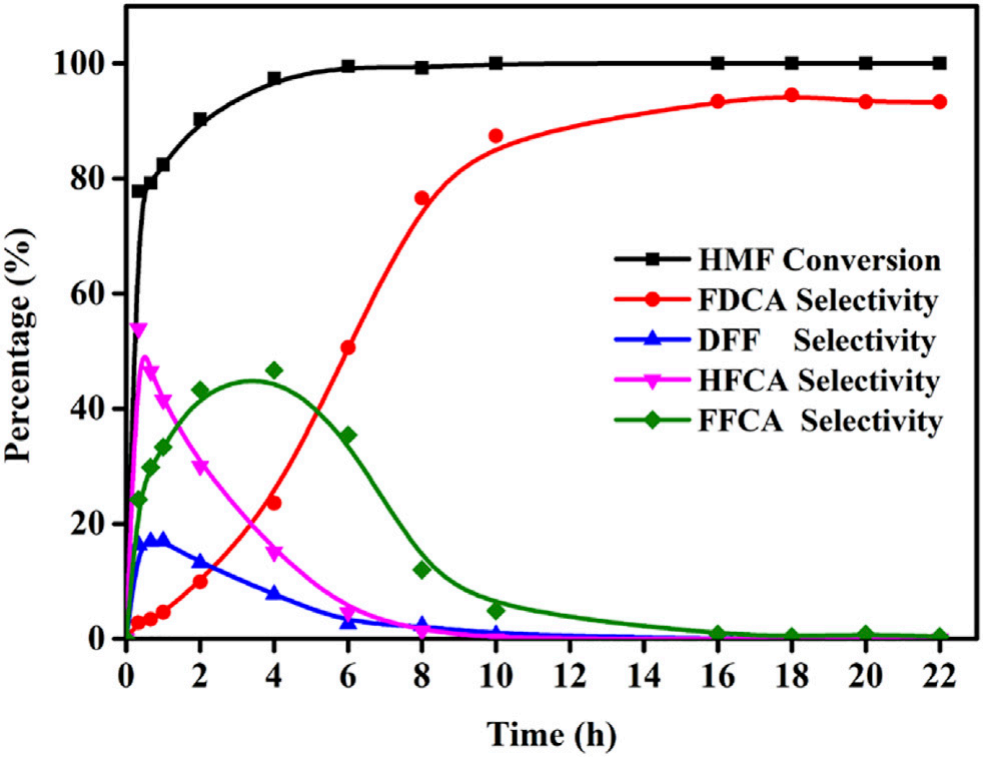
Effects of the reaction time for the oxidation of HMF over Mo-V-O. Reaction conditions:0.5 mmol HMF, 10 mmol TBHP, 30 mg catalyst, 5 ml TBA, 80°C.

Using the reaction conditions optimized above, the influence of the reaction time was then investigated (i.e., 0–22 h) to study the product distribution and selectivity changes over time. As shown in Figure 8, five oxidation products were formed, which indicates that the conversion of HMF to FDCA does not only involve the oxidation of aldehyde or hydroxyl groups individually, but also the combined oxidation of both functional groups. As a result, HMF can produce FDCA via two path ways. It was found that the HMF conversion rapidly reached 77.8% after 20 min, and then increased to almost 100.0% by 4 h. In addition, the FDCA selectivity gradually increased to 94.5% over 18 h, although variations in the DFF, HFCA, and FFCA contents were observed according to the different HMF oxidation pathways that were followed, although in these cases, an initial increase was followed by a subsequent decrease as the FDCA selectivity increased.

## 4 Reaction Kinetics of HMF Oxidation Over the Mo-V-O Catalyst

As described above, the conversion of HMF to FDCA involves both sequential and parallel reactions, which renders it difficult to estimate the intrinsic reactivity of the Mo-V-O-catalyzed process (Hayashi et al., 2019). Examination of the reaction kinetics is therefore an effective means to provide additional insight into the reaction and evaluate the catalyst performance (Davis et al., 2014; Ren et al., 2015). Initially, it was assumed that the HMF oxidation reactions were all first order reactions, and so the aforementioned activation energy was calculated using the following formulae:

$$\frac{d\left[HMF\right]}{dt}=-\left(k_{1}+k_{2}\right)\left[HMF\right]$$ (1)

$$\frac{d\left[DFF\right]}{dt}=k_{1}.[HMF]-k_{3}.[DFF]$$ (2)

$$\frac{d\left[HFCA\right]}{dt}=k_{1}.[HMF]-k_{4}.[HFCA]$$ (3)

$$\left[HMF\right]=C_{1}\cdot e^{-\left(k_{1}+k_{2}\right)t}$$ (4)

$$\left[DEF\right]=\frac{k_{1}C_{1}}{\left(k_{3}-\left(k_{1}+k_{2}\right)\right)}\cdot \left(e^{-\left(k_{1}+k_{2}\right)t}-e^{-k_{3}\cdot t}\right)$$ (5)

$$\left[HFCA\right]=\frac{k_{2}C_{1}}{\left(k_{4}-\left(k_{1}+k_{2}\right)\right)}\cdot \left(e^{-\left(k_{1}+k_{2}\right)t}-e^{-k_{4}\cdot t}\right)$$ (6)

Due to the particularity of the reaction process, the activation energy was only partially calculated. More specifically, the data calculated at different temperatures were fit to obtain the Arrhenius plot shown in Supplementary Figure S1 (Ma et al., 2019), and the apparent activation energies (E_a_) for the oxidation of HMF to DFF and HFCA were determined to be 18.17 and 4.58 kJ mol^−1^, respectively, illustrating the preference toward oxidation of the aldehyde group by Mo-V-O. Subsequently, the further oxidation reaction of DFF and HFCA were investigated (see Supplementary Figure S2), and were calculated to be 39.7 and 60.7 kJ mol^−1^, demonstrating the preferential oxidation of the alcohol group by Mo-V-O. The E_a_ results are shown in Table 1, E_1_> E_2_ and E_3_> E_4_ indicated DFF was more susceptible to further oxidation which was consistent with Figure 8 where the HFCA selectivity was higher than the DFF selectivity at the same time.

**TABLE 1:** The activation energy of the reaction.

Entry	Ea (kJ/mol)
E_1_	18.17
E_2_	4.58
E_3_	39.7
E_4_	60.7

## 5 Response Surface Methodology (RSM)

Response surface methodology (RSM) is a statistical method for solving multivariable problems by using a reasonable experimental design method and obtaining certain data through experiments. For this purpose, a multiple quadratic regression equation is used to fit the functional relationship between the factors and response values, and the optimal process parameters are determined through analysis of the regression equation (Bezerra et al., 2008; Yemi and Mazza, 2012; Gaidoumi et al., 2019; Yabalak et al., 2018). The Box-Behnken design (BBD) software, which is a quadratic response surface, has been applied to approximate a response function for experimental data that cannot be described by linear functions (Seo and Han, 2014). Hence, the optimal conditions were confirmed by the BBD using RSM which exhibited the FDCA selectivity. The BBD requires three levels of each experimental factor, which are coded as −1, 0, and +1. Thus, the FDCA selectivity was predicted using different combinations of three independent variables, as summarized in Table 2. The quadratic model that illustrates the interaction between the dependent and independent variables is as follows:

$$Y=\beta_{0}+\beta_{A}A+\beta_{B}B+\beta_{C}C+\beta_{AB}AB+\beta_{AC}AC+\beta_{BC}BC+\beta_{AA}A^{2}+\beta_{BB}B^{2}+\beta_{CC}C^{2}+\varepsilon$$ (7)

where Y is the predicted response; A, B, and C represent the three independent variables; *β*_i_ is the linear effect; *β*_ii_ is the squared effect; and *β*_ij_ is the interaction effect. *β*_0_ represents the constant and random errors.

**TABLE 2:** Experimental design of the independent variables.

Independent variables	Factors	Ranges and levels		
		−1	0	1
Time(h)	A	13	10	16
Catalyst dosage (mg)	B	25	30	35
TBHP/HMF	C	20	30	40

For the purpose of this study, three factors were considered, namely the time (A), the catalyst dosage (B), and the TBHP: HMF molar ratio (C). The tested conditions included reaction times of 10–16 h, catalyst dosages of 25–35 mg, and TBHP:HMF molar ratios of 1:20–1:40. A total of 17 experimental runs were included, as outlined in Table 3. Data analysis was performed using Design Expert 8.0.6.1(Stat-Ease., Inc., MN, United States) (Sahoo and Gupta, 2013).

**TABLE 3:** Box-Behnken design of experiments.

Entry	Factor			FDCA selectivity (%)
	Time(h)	Catalyst dosage (mg)	TBHP/HMF	
1	13 (-1)	25 (-1)	20 (-1)	77.5
2	13 (-1)	30 (0)	30 (0)	87.0
3	13 (-1)	35 (+1)	40 (+1)	88.3
4	13 (-1)	30 (0)	30 (0)	85.7
5	13 (-1)	30 (0)	30 (0)	82.9
6	10 (0)	25 (-1)	30 (0)	87.5
7	13 (-1)	30 (0)	30 (0)	89.6
8	13 (-1)	30 (0)	30 (0)	82.3
9	16 (+1)	35 (+1)	30 (0)	86.4
10	10 (0)	30 (0)	40 (+1)	87.6
11	10 (0)	30 (0)	20 (-1)	78.2
12	16 (+1)	25 (-1)	30 (0)	84.9
13	13 (-1)	35 (+1)	20 (-1)	80.4
14	16 (+1)	30 (0)	20 (-1)	85.6
15	16 (+1)	30 (0)	40 (+1)	88.9
16	13 (-1)	25 (-1)	40 (+1)	85.5
17	10 (0)	35 (+1)	30 (0)	88.2

In the quadratic model, analysis of variance (ANOVA) was used to analyze the adequacy of the developed model, and the results are presented in Table 4. Generally, the suitability of the model is confirmed by a higher Fisher’s value (F value) with a probability value (*p* value) that is as low as possible. More specifically, values of “Prob > F″ lower than 0.0500 indicating the model terms are significant. In this case, TBHP: HMF is a significant model term. In addition, the “Lack of Fit F-value” of 1.30 implies that the Lack of Fit is not significant relative to the pure error, which means the model has good predictability. Thus, the second order polynomial equation that describes the HMF yield in terms of the actual parameters is given here:

FDCA Selectivity=+66.30907+0.17917∗A+0.19750∗B+0.35750∗C (8)

**TABLE 4:** ANOVA results of the quadratic model.

Source	Sum of squares	Df	Mean square	F-value	*p*-value prob > F	
Model	112.36	3	37.45	5.27	0.0134	significant
A-Time	2.31	1	2.31	0.33	0.5782	
B-Catalyst Amount	7.80	1	7.80	1.10	0.3138	
C-TBHP/HMF	102.24	1	102.24	14.39	0.0022	
Residual	92.36	13	7.10			
Lack of fit	68.77	9	7.64	1.3	0.4299	not significant
Pure error	23.59	4	5.90			
Cor total	204.72	16				

**FIGURE 9:**
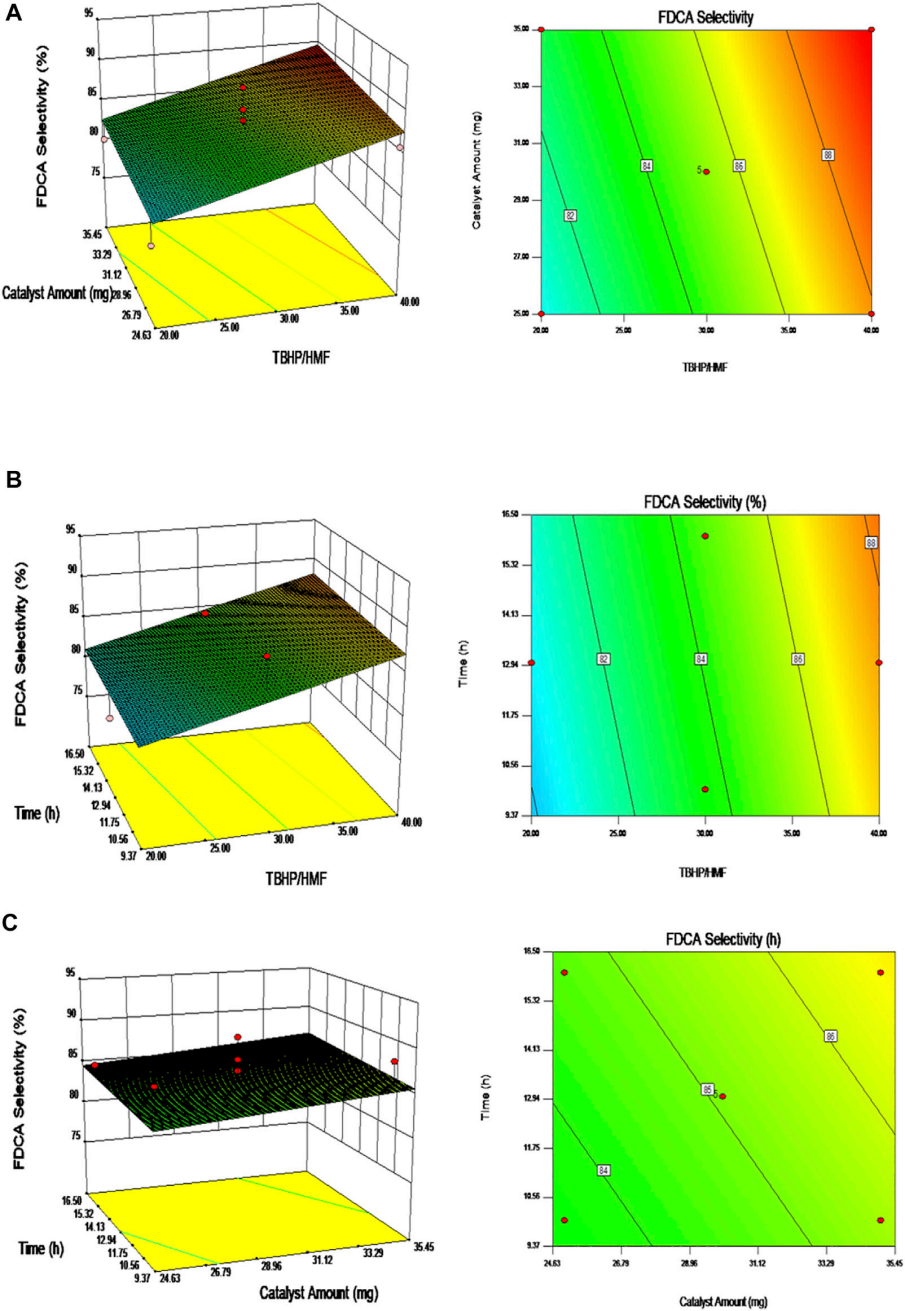
The surface-response plots on the FDCA selectivity: **(A)** Fixed Time; **(B)** Fixed catalyst amount; **(C)** Fixed TBHP/HMF molar ratio.

As shown in Figure 9, the experimental results can be presented in the form of three-dimensional response-surface plots and contour plots under fixed conditions. These plots clearly show the integrated effects of the two random variables, wherein all plots confirmed that the FDCA selectivity increased with an increase in any individual variable. Thus, according to the RSM, the selectivity toward FDCA changes upon variation in the reaction factors.

## 6 Conclusion

We herein reported the development of a simple and feasible method for preparing a catalyst for the oxidation of 5-hydroxymethylfurfural (HMF) to 2,5-furandicarboxylic acid (FDCA) in the absence of base. More specifically, a Mo-V-O catalyst was successfully prepared and was found to exhibit an excellent catalytic performance when *tert*-butyl hydroperoxide (TBHP) was employed as the sole oxidant. Under the optimized conditions determined during the course of our study, HMF was converted to FDCA with 98.2% conversion and 94.5% selectivity. Furthermore, the apparent activation energy was calculated to determine the oxidation sequence of the hydroxyl and aldehyde groups of the reactant and the intermediates. Moreover, response surface methodology was used to investigate the relationship between the catalyst loading, the TBHP/HMF molar ratio, and the reaction time. It was found that these three parameters positively affected the HMF oxidation efficiency. These results are of particular interest due to the ongoing necessity to utilize renewable resources to produce extensive commodity chemicals. Finally, considering the cost advantages of non-noble metal catalysts, further research is desirable in the oxidation of HMF to FDCA under the base-free conditions established herein.

## Data Availability

The original contributions presented in the study are included in the article/Supplementary Material, further inquiries can be directed to the corresponding author.

## References

[B1] Bezerra M. A. Santelli R. E. Oliveira E. P. Villar L. S. Escaleira L. A. (2008). Response Surface Methodology (RSM) as a Tool for Optimization in Analytical Chemistry. Talanta 76, 965–977. 10.1016/j.talanta.2008.05.019 18761143

[B2] Davis S. E. Benavidez A. D. Gosselink R. W. Bitter J. H. de Jong K. P. Davis R. J. (2014). Kinetics and Mechanism of 5-hydroxymethylfurfural Oxidation and Their Implications for Catalyst Development. J. Mol. Catal. A: Chem. 388-389, 123–132. 10.1016/j.molcata.2013.09.013

[B3] Gaidoumi A. E. Loqman A. Benadallah A. C. Bali B. E. Kherbeche A. (2019). Co(II)-pyrophyllite as Catalyst for Phenol Oxidative Degradation: Optimization Study Using Response Surface Methodology. Waste Biomass Valori. 10, 1043–1051. 10.1007/s12649-017-0117-5

[B4] Gao T. Yin Y. Zhu G. Cao Q. Fang W. (2020). Co_3_O_4_ NPs Decorated Mn-Co-O Solid Solution as Highly Selective Catalyst for Aerobic Base-free Oxidation of 5-HMF to 2,5-FDCA in Water. Catal. Today 355, 252–262. 10.1016/j.cattod.2019.03.065

[B5] Gao Z. Li C. Fan G. Yang L. Li F. (2018). Nitrogen-doped Carbon-Decorated Copper Catalyst for Highly Efficient Transfer Hydrogenolysis of 5-hydroxymethylfurfural to Convertibly Produce 2,5-dimethylfuran or 2,5-dimethyltetrahydrofuran. Appl. Catal. B: Environ. 226, 523–533. 10.1016/j.apcatb.2018.01.006

[B6] Guan J. Yang Y. Liu B. Ma Y. Yu X. Liu J. (2008). Oxidation of Isobutane over Hydrothermally Synthesized Mo-V-Te-Nb-O Mixed Oxide Catalysts. React. Kinet Catal. Lett. 95, 313–320. 10.1007/s11144-008-5387-2

[B7] Guo D. Liu X. Cheng F. Zhao W. Wen S. Xiang Y. (2020). Selective Hydrogenolysis of 5-hydroxymethylfurfural to Produce Biofuel 2, 5-dimethylfuran over Ni/ZSM-5 Catalysts. Fuel 274, 117853. 10.1016/j.fuel.2020.117853

[B8] Hayashi E. Yamaguchi Y. Kamata K. Tsunoda N. Kumagai Y. Oba F. (2019). Effect of MnO2 Crystal Structure on Aerobic Oxidation of 5-Hydroxymethylfurfural to 2,5-Furandicarboxylic Acid. J. Am. Chem. Soc. 141, 890–900. 10.1021/jacs.8b09917 30612429

[B9] Hong M. Min J. Wu S. Cui H. Zhao Y. Li J. (2019a). Metal Nitrate Catalysis for Selective Oxidation of 5-Hydroxymethylfurfural into 2,5-Diformylfuran under Oxygen Atmosphere. ACS Omega 4, 7054–7060. 10.1021/acsomega.9b00391 31459816 PMC6648045

[B10] Hong M. Wu S. Jena H. S. Li J. Ding L. Wang J. (2021). Bio-based green Solvent for Metal-free Aerobic Oxidation of 5-hydroxymethylfurfural to 2,5-diformylfural over Nitric Acid-Modified Starch. Catal. Commun. 149, 106196. 10.1016/j.catcom.2020.106196

[B11] Hong M. Wu S. Li J. T. Wang J. Wei L. F. Li K. (2019b). Aerobic Oxidation of 5-(hydroxymethyl)furfural into 2,5-diformylfuran Catalyzed by Starch Supported Aluminum Nitrate. Catal. Commun. 136, 105909. 10.1016/j.catcom.2019.105909

[B12] Lai J. Zhou S. Cheng F. Guo D. Liu X. Xu Q. (2020). Efficient and Selective Oxidation of 5-hydroxymethylfurfural into 2, 5-diformylfuran Catalyzed by Magnetic Vanadium-Based Catalysts with Air as Oxidant. Catal. Lett. 150, 1301–1308. 10.1007/s10562-019-03041-w

[B13] Lin K.-Y. A. Oh W.-D. Zheng M.-W. Kwon E. Lee J. Lin J.-Y. (2021). Aerobic Oxidation of 5-hydroxymethylfurfural into 2,5-diformylfuran Using Manganese Dioxide with Different crystal Structures: A Comparative Study. J. Colloid Interf. Sci. 592, 416–429. 10.1016/j.jcis.2021.02.030 33691223

[B14] Ma Y. Zhang T. Chen L. Cheng H. Qi Z. (2019). Self-developed Fabrication of Manganese Oxides Microtubes with Efficient Catalytic Performance for the Selective Oxidation of 5-hydroxymethylfurfural. Ind. Eng. Chem. Res. 58, 13122–13132. 10.1021/acs.iecr.9b02650

[B15] Navarro O. C. Canós A. C. Chornet S. I. (2009). Chemicals from Biomass: Aerobic Oxidation of 5-Hydroxymethyl-2-Furaldehyde into Diformylfurane Catalyzed by Immobilized Vanadyl-Pyridine Complexes on Polymeric and Organofunctionalized Mesoporous Supports. Top. Catal. 52, 304–314. 10.1007/s11244-008-9153-5

[B16] Pal P. Kumar S. Devi M. M. Saravanamurugan S. (2020). Oxidation of 5-hydroxymethylfurfural to 5-formyl Furan-2-Carboxylic Acid by Non-precious Transition Metal Oxide-Based Catalyst. J. Supercrit. Fluids 160, 104812. 10.1016/j.supflu.2020.104812

[B17] Pal P. Saravanamurugan S. (2020). Recent Advances in the Development of 5‐Hydroxymethylfurfural Oxidation with Base (Nonprecious)‐Metal‐Containing Catalysts. ChemSusChem 12, 145–163. 10.1002/cssc.201801744 30362263

[B18] Rasteiro L. F. Vieira L. H. Possato L. G. Pulcinelli S. H. Santilli C. V. Martins L. (2017). Hydrothermal Synthesis of Mo-V Mixed Oxides Possessing Several Crystalline Phases and Their Performance in the Catalytic Oxydehydration of Glycerol to Acrylic Acid. Catal. Today 296, 10–18. 10.1016/j.cattod.2017.04.006

[B19] Ren F. Zheng Y.-F. Liu X.-M. Yang Q.-Q. Zhang Q. Shen F. (2015). Thermal Oxidation Reaction Process and Oxidation Kinetics of Abietic Acid. RSC Adv. 5, 17123–17130. 10.1039/C4RA16791K

[B20] Rodikova Y. Zhizhina E. (2020). Catalytic Oxidation of 5-hydroxymethylfurfural into 2, 5-diformylfuran Using V-Containing Heteropoly Acid Catalysts. React. Kinet. Mech. Cat. 130, 403–415. 10.1007/s11144-020-01782-z

[B21] Sahoo C. Gupta A. K. (2013). KApplication of Statistical Experimental Design to Optimize the Photocatalytic Degradation of a Thiazin Dye Using Silver Ion-Doped Titanium Dioxide. J. Environ. Sci. Health A 48, 694–705. 10.1080/10934529.2013.744598 23445413

[B22] Seo Y. H. Han J.-I. (2014). Direct Conversion from Jerusalem Artichoke to Hydroxymethylfurfural (HMF) Using the Fenton Reaction. Food Chem. 151, 207–211. 10.1016/j.foodchem.2013.11.067 24423522

[B23] Tirsoaga A. El Fergani M. Nuns N. Simon P. Granger P. Parvulescu V. I. (2020). Multifunctional Nanocomposites with Non-precious Metals and Magnetic Core for 5-HMF Oxidation to FDCA. Appl. Catal. B: Environ. 278, 119309. 10.1016/j.apcatb.2020.119309

[B24] Wang F. Ueda W. (2009). Selective Oxidation of Alcohols Using Novel Crystalline Mo-V-O Oxide as Heterogeneous Catalyst in Liquid Phase with Molecular Oxygen. Catal. Today 144, 358–361. 10.1016/j.cattod.2008.12.034

[B25] Yabalak E. Görmez Ö. Gizir A. M. (2018). Subcritical Water Oxidation of Propham by H2O2 Using Response Surface Methodology (RSM). J. Environ. Sci. Health B 53, 334–339. 10.1080/03601234.2018.1431468 29431600

[B26] Yemiş O. Mazza G. (2012). Optimization of Furfural and 5-hydroxymethylfurfural Production from Wheat Straw by a Microwave-Assisted Process. Bioresour. Technol. 109, 215–223. 10.1016/j.biortech.2012.01.031 22297050

[B27] Zhang Y. F. Zheng J. Q. Zhao Y. F. Hu T. Gao Z. M. Meng C. G. (2016). Fabrication of V_2_O_5_ with Various Morphologies for High-Performance Electrochemical Capacitor. Appl. Surf. Sci. 377, 385–393. 10.1016/j.apsusc.2016.03.180

[B28] Zhao D. Su T. Wang Y. Varma R. S. Len C. (2020). Recent Advances in Catalytic Oxidation of 5-hydroxymethylfurfural. Mol. Catal. 495, 111133. 10.1016/j.mcat.2020.111133

[B29] Zhao J. Chen X. Du Y. Yang Y. Lee J.-M. (2018). Vanadium-embedded Mesoporous Carbon Microspheres as Effective Catalysts for Selective Aerobic Oxidation of 5-Hydroxymethyl-2-Furfural into 2, 5-diformylfuran. Appl. Catal. A: Gen. 568, 16–22. 10.1016/j.apcata.2018.09.015

[B30] Zhou H. Xu H. H. Liu Y. (2018). Aerobic Oxidation of 5-hydroxymethylfurfural to 2, 5-furandicarboxylic Acid over Co/Mn-Lignin Coordination Complexes-Derived Catalysts. Appl. Catal. B-environ 244, 965–973. 10.1016/j.apcatb.2018.12.046

