# Supplementary material for: Production of the 2,5-Furandicarboxylic Acid Bio-Monomer From 5-Hydroxymethylfurfural Over a Molybdenum-Vanadium Oxide Catalyst
Source: Front Chem. 2022 Mar 14;10:853112. doi: 10.3389/fchem.2022.853112 (PMC8967152; doi:10.3389/fchem.2022.853112)
Supplement: Supplementary file 1 [file DataSheet1.doc]

**Supplementary information**

# Production of the 2,5-furandicarboxylic acid bio-monomer from 5-hydroxymethylfurfural over a molybdenum-vanadium oxide catalyst

Jian Liu, Sha Wen, Fei Wang, Xiaoting Zhu, Zhijuan Zeng, Dulin Yin^*^

National & Local Joint Engineering Laboratory for New Petro-chemical Materials and Fine Utilization of Resources, Key Laboratory of the Assembly and Application of Organic Functional Molecules of Hunan Province, Hunan Normal University, Changsha 410081, China

*** Correspondence:**Corresponding Author
dulinyin@126.com


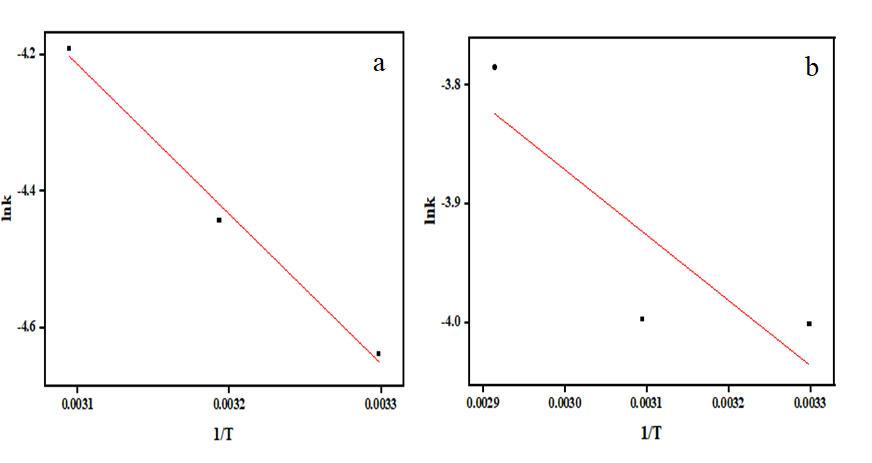


**Figure S1** Arrhenius plots obtained from data collected at different temperatures: (a) HMF oxidation to DFF, and (b) HMF oxidation to HFCA.


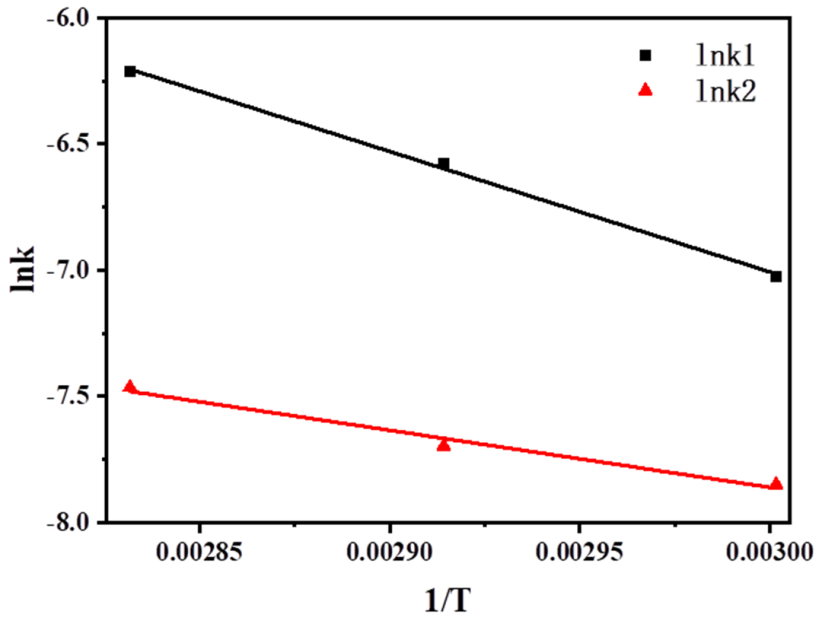


**Figure S2** Arrhenius plots obtained from data collected at different temperatures. (lnk1) DFF-to-FFCA, (lnk2) HFCA-to-FFCA.
