# Supplementary figures and images for: Production of the 2,5-Furandicarboxylic Acid Bio-Monomer From 5-Hydroxymethylfurfural Over a Molybdenum-Vanadium Oxide Catalyst
Source: Front Chem. 2022 Mar 14;10:853112. doi: 10.3389/fchem.2022.853112 (PMC8967152; doi:10.3389/fchem.2022.853112)

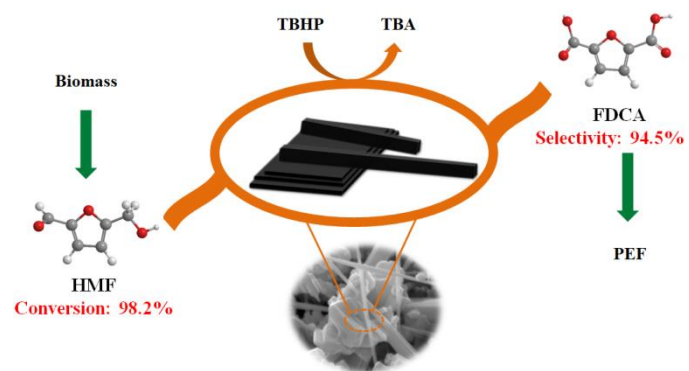

Supplement: Supplementary file 2 [file Image1.pdf]
